# Hormone Replacement Therapy: Would it be Possible to Replicate a Functional Ovary?

**DOI:** 10.3390/ijms19103160

**Published:** 2018-10-14

**Authors:** Swati Agarwal, Faisal A Alzahrani, Asif Ahmed

**Affiliations:** 1 Aston Medical Research Institute, Aston Medical School, Aston University, Birmingham B4 7ET, UK; asif.ahmed@aston.ac.uk; 2 Department of Obstetrics and Gynecology, University of Toronto, Toronto, ON M5S 3H7, Canada; 3 Department of Biochemistry, Faculty of Science, King Abdulaziz University, Jeddah 21589, Saudi Arabia, faahalzahrani@kau.edu.sa; 4 Embryonic Stem Cell Unit, King Fahad Center for Medical Research, King Abdulaziz University, Jeddah 21589, Saudi Arabia

**Keywords:** hormone replacement therapy, menopause, ovary

## Abstract

Background: Throughout history, menopause has been regarded as a transition in a woman’s life. With the increase in life expectancy, women now spend more than a third of their lives in menopause. During these years, women may experience intolerable symptoms both physically and mentally, leading them to seek clinical advice. It is imperative for healthcare providers to improve the quality of life by reducing bothersome menopausal symptoms and preventing disorders such as osteoporosis and atherosclerosis. The current treatment in the form of hormone replacement therapy (HRT) is sometimes inadequate with several limitations and adverse effects. Objective and rationale: The current review aims to discuss the need, efficacy, and limitations of current HRT; the role of other ovarian hormones, and where we stand in comparison with ovary-in situ; and finally, explore towards the preparation of an HRT model by regeneration of ovaries tissues through stem cells which can replicate a functional ovary. Search methods: Four electronic databases (MEDLINE, Embase, Web of Science and CINAHL) were searched from database inception until 26 April 2018, using a combination of relevant controlled vocabulary terms and free-text terms related to ‘menopause’, ‘hormone replacement therapy’, ‘ovary regeneration’, ‘stem cells’ and ‘ovarian transplantation’. Outcomes: We present a synthesis of the existing data on the efficacy and limitations of HRT. HRT is far from adequate in postmenopausal women with symptoms of hormone deprivation as it fails to deliver all hormones secreted by naïve ovarian tissue. Moreover, the pharmacokinetics of synthetic hormones makes them substantially different from natural ones. Not only does the number and type of hormones given in HRT matter, but the route of delivering and their release in circulation are also imperative. The hormones are delivered either orally or topically in a non-physiological uniform manner, which brings along with it several side effects. These identify the need for a hormone delivery system which replicates, integrates and reacts as per the requirement of the female body. Wider implications: The review outlines the strengths and weaknesses of HRT and highlights the potential areas for future research. There is a tremendous potential for research in this field to understand the collective roles of the various ovarian hormones and to devise an auto-regulated hormone delivery system which replicates the normal physiology. Its clinical applications can prove to be transformative for postmenopausal women helping them to lead a healthy and productive life.

## 1. Background

**Figure 1:**
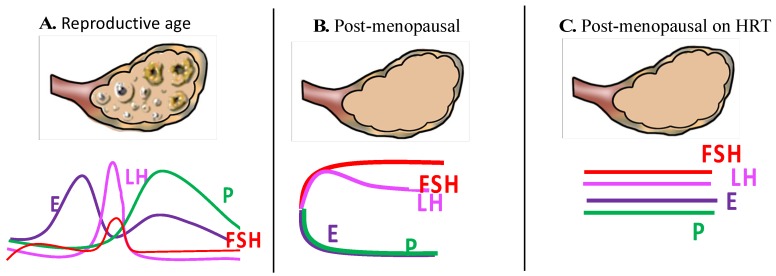
Schematic representation of the level of various ovarian hormones in reproductive age, post-menopausal and in women on HRT.

Throughout history, menopause has been regarded as a major transition in a woman’s life. With the increase in life expectancy, women now spend more than a third of their life in menopause. During these years, women experience intolerable symptoms and seek clinical advice. It is imperative for healthcare providers to improve the quality of lives by reducing bothersome menopausal symptoms and preventing disorders such as osteoporosis, atherosclerosis and coronary heart disease, dyslipidemia, and so on. However, a question that is yet to be determined is: “Is the age of natural menopause a marker of health and aging?” The onset of menopause has been considered as an ideal time to introduce preventative strategies to improve the longevity and quality of postmenopausal women’s lives. The current hormone replacement therapy (HRT), which is primarily based on estrogen and progesterone, has been prescribed over decades for the treatment of postmenopausal symptoms, and has been questioned time and again owing to its side effects and efficacy in substituting for a functional ovary. Recent developments in estrogen and progesterone-based drugs and mode of deliveries have extended the benefits of HRT; however, it is yet to be equivalent to functional ovaries of premenopausal women. Along with releasing eggs, ovaries secrete several hormones, including oestrogen, progesterone, testosterone, activin, inhibin, anti-Müllerian hormone (AMH) and insulin-like growth factor 1 (IGF-1), which have an immense significance on a woman’s physiology [1]. The secretion and functioning of these hormones are complexly integrated and intricately regulated by many positive and negative feedback loops (Figure 1). Hence, the ovary must not be regarded as a static endocrine organ, which expands at puberty and contracts with menopause, rather, it is a dynamic organ whose periodicity is measured in weeks. It has immensely beneficial effects on women’s health and is considered to be the prime attribute for lower morbidity and mortality, as evidenced in premenopausal women compared to men of the same age group. Later age at natural menopause has been found to be associated with plenty of benefits, including longevity and reduced all-cause mortality [2,3,4]. There is a decreased prevalence of atherosclerosis and cardiovascular events in women with late age at natural menopause [5,6,7,8,9]. Moreover, there are some additional benefits, such as a substantial increase in the bone density and consequently a reduction in the incidence of osteoporosis and fractures [10,11]. Nevertheless, it comes at a cost of increased risk of breast [12], ovarian [3,13] and endometrial [14] cancers. However, unlike a functional ovary, the exogenous hormones, oestrogen and progesterone, prescribed during the postmenopausal period in a stereotyped manner to replete the functioning of in situ ovary bring along several side effects.

The present review intends to discuss the need, efficacy, and limitations of current oestrogen- and progesterone-based HRT; where we stand in comparison with a functioning ovary in situ; and finally, to explore towards the preparation of an ideal HRT model which can truly replicate a functional ovary. We have evaluated the published literature on the benefits and risks associated with HRT, and the importance of other hormones secreted by the functioning premenopausal ovary which are not currently included in the HRT. We have further explored the emerging opportunities for the development of a flawless HRT model, which could help post-menopausal women to battle through this period, and which could be achieved by understanding the role, mode and regulation of all hormones secreted by functioning ovaries, improving the present oestrogen- and progesterone-based drugs and their delivery system.

## 2. Health Concerns with Menopause/Need for HRT

**Figure 2:**
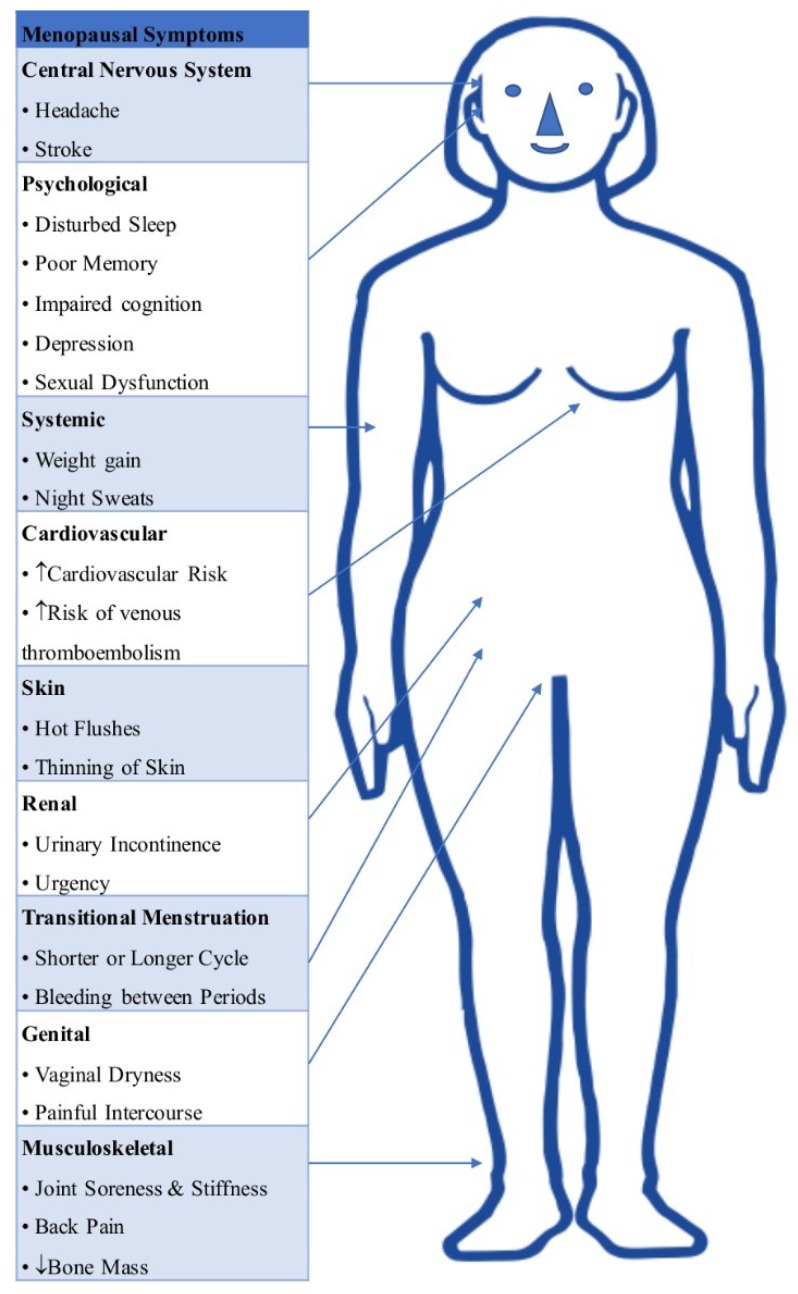
Schematic figure depicting various symptoms of menopause.

The health problems of women undergoing menopause are varied, ranging from mild mood issues to severe problems such as myocardial infarction, and are amenable to treatment to varying degrees by HRT. They are discussed in detail in the following paragraphs (Figure 2).

### 2.1. Coronary Artery Disease

Heart disease is the leading cause of death for women in the developed world [15]. Before the age of 40 years, women have a lower incidence of coronary heart disease (CHD) and myocardial infarction than men (WHO 2009). The lower incidence in premenopausal women is attributed to the favourable effect of oestrogen on the lining of the blood vessels, the endothelium [16,17]. However, following menopause, the benefit slowly fades, and women are at a similar risk of dying from cardiovascular diseases as men. Similarly, women with premature ovarian failure are at an increased risk of CHD [18]. The increased risk with HRT was unveiled by Women’s Health Initiative (WHI) and the Heart and Estrogen-progestin Replacement Study (HERS) I and HERS II studies [19,20,21]. In 2013, WHI investigators reported age as a major attribute in the benefit-risk balance following hormone use [22]. Presently, many observational studies suggest that if HRT is started immediately following menopause, it has more beneficial effects on the cardiovascular health and leads to decreased mortality. In contrast, the mortality due to heart diseases is increased if HRT is started more than 10 years after menopause or after the age of 60 years [23,24,25]. Thus, this ‘therapeutic window’ for starting HRT gives a limitation to the use of HRT in the prevention of cardiac disease [26]. The prothrombotic effects of oestrogen include a decrease in the levels of serum fibrinogen, factor VII, and antithrombin III. These effects are less pronounced in case of transdermal oestrogen but the evidence that transdermal oestrogen is safer for secondary prevention of CHD is lacking [27,28]. In addition, the choice and regimen of progesterone is important as there is some evidence that some progesterone like medroxy progesterone acetate (MPA) (used in WHI and HERS) might attenuate the cardiac benefit caused by oestrogen while norethindrone acetate or natural progesterone are not found to lessen the benefit of oestrogen. Studies have shown that combined oestrogen-progesterone therapy does not affect the incidence of peripheral arterial events [29].

### 2.2. Metabolic Effects

The remarkable effects of oestrogen on women’s metabolism, and its elusive physiological shield against metabolic syndrome in women of reproductive age making oestrogen the most likely candidate for the HRT. The deficiency of oestrogen postmenopausally causes the development of metabolic syndrome (41.5%) and hypertriglyceridemia (56.9%) in almost half of menopausal women [30,31,32]. Women with metabolic syndrome have six times increased risk of developing CHD and the underlying pathophysiology could be related to insulin resistance or central obesity [33]. HRT, instead of restoring the imbalance, further worsens some of the metabolic effects like hypertriglyceridemia, while decreasing low-density lipoprotein and increasing high-density lipoprotein levels [34]. It is notable that HRT appears to decrease the risk of diabetes mellitus type 2; however, the impact is insufficient for HRT to be recommended for its prevention [35,36].

### 2.3. Osteoporosis

Osteoporosis is widely recognised as one of the most common bone problems in elderly females. It is a generalised skeletal disorder ascribed to decreased bone mass with increased susceptibility to fractures. Seventy-five percent or more of bone mass loss has been attributed to the declining oestrogen levels during the postmenopausal period, rather than aging [37]. Oestrogen exerts a constant suppressive action on the remodelling of bone and with reduced oestrogen levels in menopause. The bone resorption is enhanced by oestrogen. The risk of sustaining a fracture in a postmenopausal woman is almost twice the lifetime probability of developing breast cancer for a woman [38,39,40].

Although HRT is not the first line treatment in postmenopausal women for the prevention or treatment of osteoporosis, WHI study revealed a significant reduction in fractures in women on HRT compared to women on placebo [41]. While, HRT is effective in osteoporosis, it is not uniformly recommended by all the major societies for its prevention [42,43,44,45].

### 2.4. Vasomotor Symptoms (VMS)

Commonly known as hot flushes or night sweats (VMS) are experienced by about 60–80% of postmenopausal women during the perimenopausal phase [46,47,48]. Most importantly, VMS severely affect women’s sense of wellbeing and quality of life [49,50]. According to a Cochrane review, there was a significant reduction in the frequency and severity of hot flushes with HRT as compared to placebo [51,52]. HRT is recommended for women with moderate to severe symptoms and no contraindications for short-term symptom relief. It can be speculated that the other ovarian hormones also play a potential role in triggering these symptoms. 

### 2.5. Vulvovaginal Atrophy (VVA)

Oestrogen deficiency results in atrophy of the vaginal mucosa, causing vaginal dryness, dyspareunia (painful intercourse), itching, vaginal stenosis and urinary incontinence. Similar to VMS, VVA is highly prevalent among postmenopausal women, with prevalence ranging from 4% in early menopause to 47% by three years after the last menses [53]. Low-dose vaginal oestrogen preparations are shown to be highly efficacious in treating VMS [54]. The American College of Obstetricians and Gynaecologists and North American Menopause Society recommend the indefinite use of low-dose vaginal therapy for as long as needed; however, there is limited evidence regarding the safety of vaginal preparations beyond one year of use [55,56]. Similarly, another study was recently published reassuring the long-term safety of vaginal oestrogen [57].

The incidence of urinary tract infections and the symptoms of overactive bladder are found to decrease with topical oestrogen therapy [54,58]. Urinary incontinence affects 26–40% of women beyond the age of 55 years [59]. It is hypothesised that oestrogen plays a vital role in the continence mechanism; however, randomised control trials (RCTs) have failed to demonstrate improvement in urinary incontinence in post-menopausal females following treatment with oestrogen [60,61,62]. In contrast, HERS reported an increase in incontinence with HRT [63]. The worsening of symptoms with exogenous administration of oestrogen is an enigma as biological oestrogen secreted by the ovary is protective, as evidenced by a lower incidence in younger women [59].

### 2.6. Cognition and Dementia

Studies have reported that dementia occurs less frequently in oestrogen users and the preventive effect is greater with increasing dose and duration of use [64,65,66]. Oestradiol replacement in rats found that it increases the activity of choline acetyltransferase in various parts of the brain related to memory and cognition [67]. Others have advocated a critical window period for starting HRT [68,69,70] for dementia. No such difference was found in studies where HRT was given to older postmenopausal females (>65 years) [71,72]. On the other hand, an increased risk of dementia was found when oestrogen was given in combination with progesterone (HRT) [73]. A Cochrane review in 2008 has refuted any benefits of HRT on cognition [74]. Another review reflected both possible beneficial and detrimental impact of oestrogen on memory in the aging brain [75]. There appears to be no conclusive study to give a definitive answer to this critical concern.

### 2.7. Sleep Disturbances

The quality of sleep deteriorates with aging [76], and menopause further contributes to it [77,78]. The Study of Women’s Health Across the Nation (SWAN) in the United States of America reported sleep disturbances in 36.6% of late perimenopausal or postmenopausal women [79]. The role of HRT is quite limited in treating sleep disorders associated with menopause.

### 2.8. Overall Effects of HRT

The ovary has an immense benefit for women’s health and is the primary organ responsible for lowering morbidity and mortality in premenopausal women compared to men of the same age group. However, the advantages gained through functioning ovaries, decline with menopause and HRT does not appear to reap the expected benefits. Moreover, HRT, when prescribed for a long duration, is associated with many side effects, some being life-threatening. Therefore, the benefits must be weighed against the risks before prescribing HRT. Though it should be noted that long-term exposure to endogenous hormones is also associated with increased risks. Further research is required not only to validate these findings but also develop a novel hormone replacement model which can replicate the natural functioning of ovary without causing the adverse effects.

## 3. Current Preparations of HRT in Clinical Practice

Current HRT mainly consists of oestrogen and progesterone in various preparations differing in their dosages and routes of administration, each having its benefits and risks (Table 1). However, with HRT, the menopausal symptoms can only be partially relieved, as present therapy fails to replicate the natural functioning of an ovary. The relative alleviation of menopausal symptoms is primarily due to oestrogen. The oestrogen only therapy is prescribed for a hysterectomised woman, but in a woman with an intact uterus, progestin is added to combat the increased risk of endometrial hyperplasia and even carcinoma.

Natural oestrogens are available in various plant sources like soy, but their efficacy is unclear. The modified oestrogens are called semisynthetic and include conjugated oestrogens (conjugated equine oestrogen, oestradiol valerate and oestrone sulphate) and micronized oestradiol. Conjugated equine oestrogen is prepared from the urine of pregnant mares and is composed of 50–60% oestrone sulphate along with other equine oestrogens like equilin and 17-dihydroequilin. Micronisation of oestradiol results in higher blood levels of oestrogen, but it is rapidly metabolised to less active forms like oestrone. Synthetic oestrogens such as ethinyl oestradiol have better potency and extended half-life.

Based on present evidence, physicians have unsuccessfully tried to tailor the HRT prescription as per the symptoms of menopausal women. Women are informed that their prescription for HRT has been individualised with the type and dosage of preparation being determined according to their specific requirements. However, in reality, there are no means of establishing the amount of hormones needed by a woman’s body to combat her postmenopausal symptoms. This is because the ovarian physiology is dynamic and the blood levels of hormones could be misleading since every woman develops symptoms at different hormone levels with varying severity.

### Bio-Identical HRT (BHRT)

Over the last two decades, many people are attracted by the idea of bio-identical hormones (BHRT). Bio-identical hormones are derived from plant extracts and chemically modified in the laboratory so as to be identical in molecular structure to natural female hormones [80]. There have been attempts to promote BHRT stating that these hormones are natural and, therefore, superior to synthetic HRT. Nevertheless, studies have failed to prove BHRT to be superior to conventional HRT [81,82,83].

## 4. Adverse Effects of HRT

It has been established that HRT is far from adequate in treating women with menopausal symptoms [52,84,85,86]. Moreover, it is associated with many unwanted and serious adverse effects, which are discussed subsequently. It is, however, important to note that these risks are also associated with delayed menopause.

### 4.1. Venous Thromboembolism (VTE) and Stroke

Venous thromboembolism is characterised by the formation of thrombus in deep veins of legs, which can embolise to the pulmonary circulation and even cause death. HRT increases the risk of VTE by 2-fold, mostly in the first and second years of treatment [87]. Studies have shown that transdermal oestrogen is associated with a lower risk while progesterone only HRT is not associated with an increased risk of VTE [88,89]. There is a wealth of literature showing the increased incidence of stroke in women on either oestrogen only or oestrogen-progestin HRT [90,91,92]. The current users of transdermal or oral HRT have a higher risk of stroke than non-users [93]. Hence, caution must be exercised in prescribing HRT to women with risk factors for stroke or VTE. The contention remains: why does oestrogen in HRT increase the risk of VTE while the presence of a functioning ovary does not?

### 4.2. Gynaecological Cancers

There is an increased risk of breast cancer in users of present HRT [94]. Recent studies have reported a hazard ratio of 1.24 to 2.74 in current users after 2 to 5 years of HRT use, which increases further with increasing duration of use [95,96].

A woman’s risk of having ovarian cancer during her lifetime is 1–1.5% and dying from the same disease is almost 0.5% [97]. A recent meta-analysis has revealed a relative risk of 1.43 in hormone users compared to non-users [98]. They reported increased risk, even with a limited period of use (less than five years), and the risk, although it declines, persists even ten years after discontinuing HRT. Women on unopposed oestrogen only therapy have a non-significant decrease in the incidence of breast cancer with a definite increased risk of endometrial cancer [22].

Endometrial carcinoma is the most common malignancy of the female genital tract, with 2–3% of women developing the cancer in their lifetime [99]. The risk increases manifold by taking unopposed oestrogen therapy; however, the threat of the cancer is almost entirely eliminated by the addition of progestins in a continuous fashion [100,101,102].

## 5. Caveats with HRT

**Figure 3:**
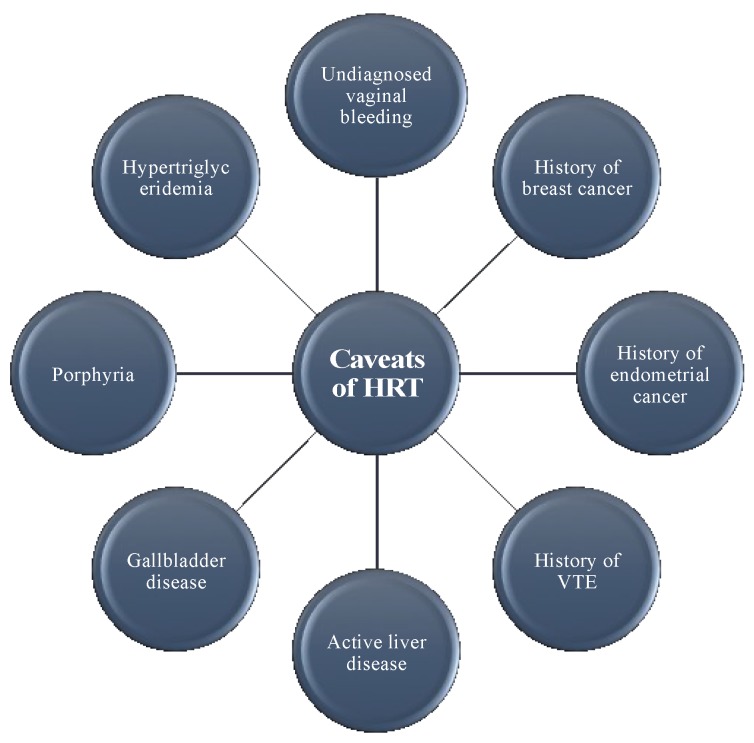
Relative contraindications to HRT.

There are many relative contraindications to HRT, although no absolute contraindications exist [103]. On the contrary, the presence of a normal functioning ovary in a woman causes less harm (if any) while offering plenty of benefits. There are hardly any indications for removing it. The contraindications of HRT are outlined in Figure 3.

## 6. The Role of Other Hormones Secreted by the Ovary

A functional ovary secretes several hormones, which play an essential role in the maintenance of women’s health and well-being; however, they are not a part of the current HRT regimens. The advancements in science and technology have helped us in identifying and isolating many of them. The ovarian hormones can be divided into two broad categories on the basis of their chemical structure. The first group is formed by the steroid hormones, which include oestrogens, androgens and progestins. The next group is the peptide hormones, which includes AMH, inhibin, activin, follistatin, IGF, relaxin, transforming growth factor-β (TGF-β), fibroblast growth factor (FGF), epidermal growth factor (EGF) and the oocyte-derived proteins GDF-9 and BMP-15. The levels of activin increase after menopause, follistatin concentrations remains the same [104,105] and the levels of all other hormones decrease. Most of them are released into the circulation and can be detected systemically. Factors such as IGF, GDF-9 and BMP-15 are released locally and act to promote follicular growth [1].

### 6.1. Androgens

In premenopausal women, levels of free testosterone are found to correlate directly with libido [106]. After menopause, though the androgen production continues, its levels are much lower than the women of the reproductive age group. Supplementation of androgen has been found to increase not only the sexual desire but also improve the psychological well-being [107,108]. Along with that, androgen boosts have helped postmenopausal females to increase bone density more than oestrogen alone. However, the benefits of adding androgens must be weighed against their undesirable effects like hirsutism and hypercholesterolemia [109,110].

### 6.2. Anti-Müllerian Hormone

Anti-Müllerian hormone (AMH also called Müllerian inhibiting substance (MIS)) is secreted in the ovary by the granulosa cells of preantral and small antral follicles [111]. It regulates the follicular development in an autocrine and paracrine manner. Several studies on AMH have found that it inhibits the proliferation of all gynaecological cancers expressing MIS receptor such as cervical, endometrial, ovarian and breast cancers [112,113,114,115]. MIS inhibits the cancer cells by interfering with cell cycle progression and inducing apoptosis in them. The lack of AMH after menopause could be one of the contributors to the increased incidence of gynaecological carcinomas postmenopausally and its supplementation could have a preventable role in their development. It could also be contemplated that AMH may play a possible role in treating gynaecological conditions like endometriosis and adenomyosis. Pepin et al., have recently used adeno-associated virus (AAV9) to deliver MIS in mice which significantly inhibited the growth of ovarian cancer cells [116]. Papakostas et al., developed a novel and efficient way of producing highly pure and biologically active internally labelled form of MIS [117]. This provides a ray of hope that incorporating AMH in HRT in the near future could make it better and closer to a functional ovary.

### 6.3. Inhibin

Inhibin is secreted by the granulosa cells of small antral follicles during the follicular phase and helps in folliculogenesis [111]. It has been found that inhibin knockout mice have a higher susceptibility to developing tumours of gonads and adrenals. This suggests a potential tumour suppressive effect of inhibin in the body [118,119]. Recombinant human inhibin has long been synthesised [120] and its addition to HRT may prove beneficial. There have been no studies done thus far; however, there is a potential for research on the topic.

### 6.4. Insulin-Like Growth Factor

IGFs are single-chain polypeptides, which resemble insulin in structure and function and are secreted by granulosa cells in response to follicular stimulating hormone (FSH). In humans, IGF-II is more active in the embryonic period while IGF-I postnatally [1]. The ovary produces high amounts of IGF-I, next only to the liver. IGF along with TGF-β is required for maintaining healthy bone mass. Recombinant human IGF-I has been synthesised in the laboratory, and there are promising studies regarding its use in cardiovascular diseases [121] and diabetes mellitus [122]. It would also be worthwhile conducting further studies to explore the potential effects of supplementing IGF in the postmenopausal state.

### 6.5. Relaxin

Relaxin is a pleiotropic hormone secreted by ovary with a role in a multitude of physiological processes besides reproduction and pregnancy. It has positive effects on almost every organ system of the body, including the brain, heart and kidney. It has a central vasopressor effect and anti-ischemic effect on brain and myocardium [123,124]. It has a relaxant effect on the vasculature mediated by nitric oxide secreted from the endothelial cells [125,126,127]. Moreover, it induces angiogenesis and inhibits the activation of inflammatory cells thus playing a key role in the prevention of CHD [128]. It is found to impede myocardial damage induced by ischaemia and reperfusion [129,130,131]. It inhibits platelet aggregation [132] and neutrophil activation [133,134]. The loss of protection against CHD in postmenopausal women is partly attributed to the loss of relaxin. Relaxin is anti-asthmatic [135,136] and promotes ciliary beating in the respiratory pathways [137]. Recombinant human relaxin (Serelaxin) has been successfully tried in patients with acute heart failure, and resulted in reduced mortality, symptom relief and improvement in clinical outcomes [138,139].

## 7. Limitations of Current HRT

Hormone replacement therapy is undoubtedly beneficial in postmenopausal women with symptoms of hormone deprivation, but it has its own limitations. Although the ovary secretes a multitude of hormones, only two of them, oestrogen and progesterone, are replaced by HRT. Also, the pharmacokinetics of the synthetic hormones makes them substantially different from the natural ones. Not only does the number and type of hormones given in HRT matter, but the route of delivering these hormones so as to resemble physiologically is also imperative. The oral preparations of oestrogen and progestin have a significant first-pass metabolism, which decreases the bioavailability of the hormones. Once oestrogen is metabolised in the liver, it gets converted to oestrone sulphate (a weak oestrogen), and subsequently, only 10% of it reaches the circulation [140]. Orally administered oestrogen also increases sex hormone binding globulin (SHBG) levels [141,142], which further reduces the bioavailability, not only of the exogenous hormone but also of the residual endogenous one. Although HRT could, to some extent, compensate for the loss of ovarian hormone production, the delivery of constant amount via pharmacological means leads to substantially higher concentrations of the hormones, causing various side effects. In the body, the levels of different hormones secreted by the ovary vary not only with the phase but also within each day of the menstrual cycle, regulated by various feedback mechanisms.

## 8. Emerging Opportunities

### 8.1. Cell-Based Hormone Delivery

An approach to providing all the ovarian hormones physiologically without causing any side effects would be cell-based hormone delivery. This concept of designing an artificial ovary involves dissecting all the cells in the ovary and subsequently amalgamating them. This would unveil the contribution of each component of the ovary and may prove clinically useful in devising an optimal HRT. Though several types of research have been undertaken, the complex, cyclical and dynamic functioning of the ovary makes the formation of an artificial ovary a tough task. In a study, theca and granulosa cells, after being isolated, were seeded into micro-moulded gels and self-assembled into complex 3D microtissue [143]. The oocytes thus created from this construct of artificial ovary were functional and could be used to replace the lost ovarian function in postmenopausal women, as they can secrete hormones in response to gonadotropins.

Sittadjody et al., devised an in vitro tissue-based technique of hormone delivery that used a functional construct manufactured utilising encapsulation technology [144]. They consolidated theca and granulosa cells isolated from rat ovaries to form the native follicular structure using multi-layered alginate microcapsules. The encapsulated cells were viable and secreted adequate levels of oestradiol, progesterone, activin and inhibin in response to FSH and luteinising hormone (LH). This suggested that the multilayer microcapsules could function as a potential tissue-engineered endocrine ovary by synchronizing with the innate hypothalamic-pituitary-ovarian (HPO) axis. The same authors also suggested that the addition of a layer of poly l-ornithine around the theca cells will protect the construct from the host immune system and enable its in vivo use as a graft in postmenopausal women. The cell encapsulation technique may prove to be efficient in delivering the cultured cells into the body. This opens a whole new platform for further research in administering HRT. A similar experiment done in ovariectomized mice using granulosa cells grown on microcarriers, enclosed with theca cells in alginate-chitosan-alginate microcapsules have shown normal levels of serum estradiol and progesterone [145]. Another group has successfully used allografted microencapsulated ovarian cells in vivo to prevent the risk of osteoporosis after ovariectomy in mice [146]. The tissue engineering approaches might prove promising to provide endogenous female hormones in symptomatic post-menopausal females.

### 8.2. Brain-Selective Oestrogen Therapy

Recently, Merchenthaler et al. discovered that oral administration of a human oestrogen precursor—10β,17β-dihydroxyestra-1,4-dien-3-one (DHED) alleviates hot flushes and restores the oestrogen deprivation-induced loss of diurnal rhythm in rat models of thermoregulatory dysfunction of the brain [147,148]. Oestrogen is selectively generated in the brain, independent of the route of administration, and thus avoids the detrimental effects of the hormone exposure in the rest of the body, including the uterus, and circumventing the need for progesterone supplementation. However, further studies are required in the field before it can be clinically applied. 

### 8.3. Ovarian Tissue Transplantation

Furthermore, studies conducted using mice models have shown that transplantation of young ovaries to old mice increases their lifespan [149,150]. The same authors also found that mice that had ceased cycling before ovarian transplantation showed an increase in lifespan compared with mice still cycling at transplantation. Shikanov et al., transplanted ovarian cortical tissue in mice. The cortical tissue was vitrified, thawed and then encapsulated in fibrin-alginate hydrogels. It was loaded with vascular endothelial growth factor (VEGF) and then placed the tissue in the bursal sac [151,152]. The biomaterial supported cell infiltration and thus promoted the integration of the graft with the host. VEGF enhanced angiogenesis with vessel growth primarily from the bursa and thus, they obtained a high degree of graft revascularisation. Few studies have also been conducted in humans, transplanting young healthy ovaries using cortical grafting technique from premenopausal monozygotic twins discordant for premature ovarian failure and successful fertility outcomes were reported [153]. There was a case reported in which a woman with Hodgkin’s lymphoma was auto-transplanted her own ovary heterotopically subcutaneously in the abdomen after receiving hematologic stem cell transplantation. She successfully regained her fertility and had three babies [154]. The technique can be used in a postmenopausal woman by removing and cryopreserving one of her ovaries after she completes the childbearing process and then auto-transplanting the ovarian tissue at menopause. It is too early to say whether the benefits of such techniques outweigh the risks. This area needs to be studied further and could prove to be a breakthrough in the treatment of postmenopausal women.

## 9. Conclusion

The review outlines the strengths and weaknesses of HRT and highlights the potential areas for future research. The inadequate symptom relief and side effects associated with current HRT continues to set new riddles and opens a whole era to explore options to reformulate the current HRT. An ideal HRT should supplement all the hormones secreted by a functional ovary in a physiological manner without any side effects. There is tremendous potential for research in this field to understand the collective roles of the various ovarian hormones and to devise an auto-regulated hormone delivery system which replicates the normal physiology. Its clinical applications can prove to be transformative for postmenopausal women helping them to lead a healthy and productive life.

## Figures and Tables

**Table 1:** Different preparations of oestrogen and progesterone.

Hormone	Preparation
Oral Oestrogen	Conjugated oestrogen Ethinyloestradiol Esterified oestrogens 17β-oestradiol
Transdermal Oestrogen	17β-oestradiol patch 17β-oestradiol gel 17β-oestradiol emulsion 17β-oestradiol spray
Vaginal Oestrogen	17β-oestradiol cream Conjugated oestrogen cream 17β-oestradiol ring 17β-oestradiol tablet
Oral Progestogen	Medroxyprogesterone acetate Norethindrone acetate Megestrol acetate Drosperinone Micronised progesterone
Transdermal Progestogen	Norethindrone acetate Levonorgoestrel
Progestin: Intrauterine System	Levonorgoestrel IUS

## References

[1] Jameson J.L., de Kretser D.M., Marshall J.C., De Groot L.J. (2013). Endocrinology Adult and Pediatric: Reproductive Endocrinology.

[2] Jacobsen B.K., Heuch I., Kvale G. (2003). Age at natural menopause and all-cause mortality: A 37-year follow-up of 19,731 Norwegian women. Am. J. Epidemiol..

[3] Ossewaarde M.E., Bots M.L., Verbeek A.L.M., Peeters P.H.M., van der Graaf Y., Grobbee D.E., van der Schouw Y.T. (2005). Age at menopause, cause-specific mortality and total life expectancy. Epidemiology.

[4] Gold E.B. (2011). The Timing of the Age at which Natural Menopause Occurs. Obstet. Gynecol. Clin. N. Am..

[5] De Kleijn M.J.J., van der Schouw Y.T., Verbeek A.L.M., Peeters P.H.M., Banga J.-D., van der Graaf Y. (2002). Endogenous estrogen exposure and cardiovascular mortality risk in postmenopausal women. Am. J. Epidemiol..

[6] Cui R., Iso H., Toyoshima H., Date C., Yamamoto A., Kikuchi S., Kondo T., Watanabe Y., Koizumi A., Inaba Y. (2006). Relationships of age at menarche and menopause, and reproductive year with mortality from cardiovascular disease in Japanese postmenopausal women: The JACC study. J. Epidemiol..

[7] Atsma F., Bartelink M.-L.E.L., Grobbee D.E., van der Schouw Y.T. (2006). Postmenopausal status and early menopause as independent risk factors for cardiovascular disease: A meta-analysis. Menopause.

[8] Lisabeth L.D., Beiser A.S., Brown D.L., Murabito J.M., Kelly-Hayes M., Wolf P.A. (2009). Age at natural menopause and risk of ischemic stroke: The Framingham heart study. Stroke.

[9] Parashar S., Reid K.J., Spertus J.A., Shaw L.J., Vaccarino V. (2010). Early menopause predicts angina after myocardial infarction. Menopause.

[10] Parazzini F., Bidoli E., Franceschi S., Schinella D., Tesio F., La Vecchia C., Zecchin R. (1996). Menopause, menstrual and reproductive history, and bone density in northern Italy. J. Epidemiol. Community Health.

[11] Van Der Voort D.J.M., van Der Weijer P.H.M., Barentsen R. (2003). Early menopause: Increased fracture risk at older age. Osteoporos. Int..

[12] Monninkhof E.M., van der Schouw Y.T., Peeters P.H. (1999). Early age at menopause and breast cancer: Are leaner women more protected? A prospective analysis of the Dutch DOM cohort. Breast Cancer Res. Treat..

[13] Franceschi S., La Vecchia C., Booth M., Tzonou A., Negri E., Parazzini F., Trichopoulos D., Beral V. (1991). Pooled analysis of 3 European case-control studies of ovarian cancer: II. Age at menarche and at menopause. Int. J. Cancer.

[14] Xu W.-H., Xiang Y.-B., Ruan Z.-X., Zheng W., Cheng J.-R., Dai Q., Gao Y.-T., Shu X.-O. (2004). Menstrual and reproductive factors and endometrial cancer risk: Results from a population-based case-control study in urban Shanghai. Int. J. Cancer.

[15] WHO (2009). World Health Statistics 2009.

[16] Felty Q. (2006). Estrogen-induced DNA synthesis in vascular endothelial cells is mediated by ROS signaling. BMC Cardiovasc. Disord..

[17] Chen S.J., Li H., Durand J., Oparil S., Chen Y.F. (1996). Estrogen reduces myointimal proliferation after balloon injury of rat carotid artery. Circulation.

[18] Shuster L.T., Rhodes D.J., Gostout B.S., Grossardt B.R., Rocca W.A. (2010). Premature menopause or early menopause: Long-term health consequences. Maturitas.

[19] Manson J.E., Hsia J., Johnson K.C., Rossouw J.E., Assaf A.R., Lasser N.L., Trevisan M., Black H.R., Heckbert S.R., Detrano R. (2003). Estrogen plus progestin and the risk of coronary heart disease. N. Engl. J. Med..

[20] Hulley S., Furberg C., Barrett-Connor E., Cauley J., Grady D., Haskell W., Knopp R., Lowery M., Satterfield S., Schrott H. (2002). Noncardiovascular disease outcomes during 6.8 years of hormone therapy: Heart and Estrogen/progestin Replacement Study follow-up (HERS II). JAMA.

[21] Hulley S., Grady D., Bush T., Furberg C., Herrington D., Riggs B., Vittinghoff E. (1998). RAndomized trial of estrogen plus progestin for secondary prevention of coronary heart disease in postmenopausal women. JAMA.

[22] Manson J.E., Chlebowski R.T., Stefanick M.L., Aragaki A.K., Rossouw J.E., Prentice R.L., Anderson G., Howard B.V., Thomson C.A., LaCroix A.Z. (2013). Menopausal hormone therapy and health outcomes during the intervention and extended poststopping phases of the Women’s Health Initiative randomized trials. JAMA.

[23] Manson J.E., Allison M.A., Rossouw J.E., Carr J.J., Langer R.D., Hsia J., Kuller L.H., Cochrane B.B., Hunt J.R., Ludlam S.E. (2007). Estrogen therapy and coronary-artery calcification. N. Engl. J. Med..

[24] Maggio M., Ceda G.P., Lauretani F., Bandinelli S., Ruggiero C., Guralnik J.M., Metter E.J., Ling S.M., Paolisso G., Valenti G. (2009). Relationship between higher estradiol levels and 9-year mortality in older women: The Invecchiare in Chianti study. J. Am. Geriatr. Soc..

[25] Schierbeck L.L., Rejnmark L., Tofteng C.L., Stilgren L., Eiken P., Mosekilde L., Køber L., Jensen J.-E.B. (2012). Effect of hormone replacement therapy on cardiovascular events in recently postmenopausal women: Randomised trial. BMJ.

[26] Rossouw J.E., Prentice R.L., Manson J.E., Wu L., Barad D., Barnabei V.M., Ko M., LaCroix A.Z., Margolis K.L., Stefanick M.L. (2007). Postmenopausal hormone therapy and risk of cardiovascular disease by age and years since menopause. JAMA.

[27] Clarke S.C., Kelleher J., Lloyd-Jones H., Slack M., Schofiel P.M. (2002). A study of hormone replacement therapy in postmenopausal women with ischaemic heart disease: The Papworth HRT atherosclerosis study. BJOG.

[28] Brosnan J.F., Sheppard B.L., Norris L.A. (2007). Haemostatic activation in post-menopausal women taking low-dose hormone therapy: Less effect with transdermal administration?. Thromb. Haemostasis.

[29] Hsia J., Criqui M.H., Rodabough R.J., Langer R.D., Resnick H.E., Phillips L.S., Allison M., Bonds D.E., Masaki K., Caralis P. (2004). Estrogen plus progestin and the risk of peripheral arterial disease: The Women’s Health Initiative. Circulation.

[30] Chedraui P., Hidalgo L., Chavez D., Morocho N., Alvarado M., Huc A. (2007). Quality of life among postmenopausal Ecuadorian women participating in a metabolic syndrome screening program. Maturitas.

[31] Gurka M.J., Vishnu A., Santen R.J., DeBoer M.D. (2016). Progression of Metabolic Syndrome Severity during the Menopausal Transition. J. Am. Heart Assoc..

[32] Ben Ali S., Belfki-Benali H., Aounallah-Skhiri H., Traissac P., Maire B., Delpeuch F., Achour N., Ben Romdhane H. (2014). Menopause and metabolic syndrome in tunisian women. BioMed Res. Int..

[33] Wilson P.W., Kannel W.B., Silbershatz H., D’Agostino R.B. (1999). Clustering of metabolic factors and coronary heart disease. Arch. Intern. Med..

[34] Miller V.T., LaRosa J., Barnabei V., Kessler C., Levin G., Smith-Roth A., Griffin M., Stoy D.B., Bush T., Zacur H. (1995). Effects of estrogen or estrogen/progestin regimens on heart disease risk factors in postmenopausal women. The Postmenopausal Estrogen/Progestin Interventions (PEPI) Trial. The Writing Group for the PEPI Trial. JAMA.

[35] Kanaya A.M., Herrington D., Vittinghoff E., Lin F., Grady D., Bittner V., Cauley J.A., Barrett-Connor E. (2003). Glycemic effects of postmenopausal hormone therapy: The Heart and Estrogen/progestin Replacement Study. A randomized, double-blind, placebo-controlled trial. Ann. Intern. Med..

[36] Margolis K.L., Bonds D.E., Rodabough R.J., Tinker L., Phillips L.S., Allen C., Bassford T., Burke G., Torrens J., Howard B. (2004). V Effect of oestrogen plus progestin on the incidence of diabetes in postmenopausal women: Results from the Women’s Health Initiative Hormone Trial. Diabetologia.

[37] Cenci S., Toraldo G., Weitzmann M.N., Roggia C., Gao Y., Qian W.P., Sierra O., Pacifici R. (2003). Estrogen deficiency induces bone loss by increasing T cell proliferation and lifespan through IFN-gamma-induced class II transactivator. Proc. Natl. Acad. Sci. USA.

[38] Ho-Pham L.T., Nguyen N.D., Vu B.Q., Pham H.N., Nguyen T.V. (2009). Prevalence and risk factors of radiographic vertebral fracture in postmenopausal Vietnamese women. Bone.

[39] Hernlund E., Svedbom A., Ivergard M., Compston J., Cooper C., Stenmark J., McCloskey E.V., Jonsson B., Kanis J.A. (2013). Osteoporosis in the European Union: Medical management, epidemiology and economic burden. A report prepared in collaboration with the International Osteoporosis Foundation (IOF) and the European Federation of Pharmaceutical Industry Associations (EFPIA). Arch. Osteoporos..

[40] Eastell R., O’Neill T.W., Hofbauer L.C., Langdahl B., Reid I.R., Gold D.T., Cummings S.R. (2016). Postmenopausal osteoporosis. Nat. Rev. Dis. Primers.

[41] Cauley J., Robbins J., Chen Z., Al E. (2003). Effects of estrogen plus progestin on risk of fracture and bone mineral density: The women’s health initiative randomized trial. JAMA.

[42] Mosca L., Benjamin E.J., Berra K., Bezanson J.L., Dolor R.J., Lloyd-Jones D.M., Newby L.K., Pina I.L., Roger V.L., Shaw L.J. (2011). Effectiveness-based guidelines for the prevention of cardiovascular disease in women—2011 update: A guideline from the american heart association. Circulation.

[43] ACOG (2014). ACOG Practice Bulletin No. 141: Management of menopausal symptoms. Obstet. Gynecol..

[44] NAMS (2017). The 2017 hormone therapy position statement of The North American Menopause Society. Menopause.

[45] Grossman D.C., Curry S.J., Owens D.K., Barry M.J., Davidson K.W., Doubeni C.A., Epling J.W.J., Kemper A.R., Krist A.H., Kurth A.E. (2017). Hormone Therapy for the Primary Prevention of Chronic Conditions in Postmenopausal Women: US Preventive Services Task Force Recommendation Statement. JAMA.

[46] Kronenberg F. (1990). Hot Flashes: Epidemiology and Physiologya. Ann. N. Y. Acad. Sci..

[47] Freeman E.W., Sammel M.D., Sanders R.J. (2014). Risk of long-term hot flashes after natural menopause: Evidence from the Penn Ovarian Aging Study cohort. Menopause.

[48] Avis N.E., Crawford S.L., Greendale G., Bromberger J.T., Everson-Rose S.A., Gold E.B., Hess R., Joffe H., Kravitz H.M., Tepper P.G. (2015). Duration of menopausal vasomotor symptoms over the menopause transition. JAMA Intern. Med..

[49] Thurston R.C., Matthews K.A., Chang Y., Santoro N., Barinas-Mitchell E., von Känel R., Landsittel D.P., Jennings J.R. (2016). Changes in heart rate variability during vasomotor symptoms among midlife women. Menopause.

[50] Thurston R.C., Johnson B.D., Shufelt C.L., Braunstein G.D., Berga S.L., Stanczyk F.Z., Pepine C.J., Bittner V., Reis S.E., Thompson D.V. (2017). Menopausal symptoms and cardiovascular disease mortality in the Women’s Ischemia Syndrome Evaluation (WISE). Menopause.

[51] MacLennan A., Lester S., Moore V. (2001). Oral oestrogen replacement therapy versus placebo for hot flushes. Cochrane Database Syst. Rev..

[52] Abdi F., Mobedi H., Mosaffa N., Dolatian M., Ramezani Tehrani F. (2016). Hormone Therapy for Relieving Postmenopausal Vasomotor Symptoms: A Systematic Review. Arch. Iran. Med..

[53] Dennerstein L., Dudley E.C., Hopper J.L., Guthrie J.R., Burger H.G. (2000). A prospective population-based study of menopausal symptoms. Obstet. Gynecol..

[54] Suckling J., Lethaby A., Kennedy R. (2006). Local oestrogen for vaginal atrophy in postmenopausal women. Cochrane Database of Syst. Rev..

[55] (2004). American College of Obstetricians and Gynecologists Women’s Health Care Physicians Sexual dysfunction. Obstet. Gynecol..

[56] The North American Menopause Society (2013). Management of symptomatic vulvovaginal atrophy: 2013 position statement of The North American Menopause Society. Menopause.

[57] Crandall C.J., Hovey K.M., Andrews C.A., Chlebowski R.T., Stefanick M.L., Lane D.S., Shifren J., Chen C., Kaunitz A.M., Cauley J.A. (2018). Breast cancer, endometrial cancer, and cardiovascular events in participants who used vaginal estrogen in the Women’s Health Initiative Observational Study. Menopause.

[58] Santen R.J., Allred D.C., Ardoin S.P., Archer D.F., Boyd N., Braunstein G.D., Burger H.G., Colditz G.A., Davis S.R., Gambacciani M. (2010). Postmenopausal hormone therapy: An Endocrine Society scientific statement. J. Clin. Endocrinol. Metab..

[59] Hannestad Y.S., Rortveit G., Sandvik H., Hunskaar S. (2000). A community-based epidemiological survey of female urinary incontinence:: The Norwegian EPINCONT Study. J. Clin. Epidemiol..

[60] Ouslander J.G., Greendale G.A., Uman G., Lee C., Paul W., Schnelle J. (2001). Effects of oral estrogen and progestin on the lower urinary tract among female nursing home residents. J. Am. Geriatr. Soc..

[61] Jackson S., Shepherd A., Brookes S., Abrams P. (1999). The effect of oestrogen supplementation on post-menopausal urinary stress incontinence: A double-blind placebo-controlled trial. Br. J. Obstet. Gynaecol..

[62] Cardozo L., Rekers H., Tapp A., Barnick C., Shepherd A., Schussler B., Kerr-Wilson R., van Geelan J., Barlebo H., Walter S. (1993). Oestriol in the treatment of postmenopausal urgency: A multicentre study. Maturitas.

[63] Grady D., Applegate W., Bush T., Furberg C., Riggs B., Hulley S.B. (1998). Heart and Estrogen/progestin Replacement Study (HERS): Design, methods, and baseline characteristics. Control. Clin. Trials.

[64] Paganini-Hill A., Henderson V.W. (1994). Estrogen deficiency and risk of Alzheimer’s disease in women. Am. J. Epidemiol..

[65] Tang M.X., Jacobs D., Stern Y., Marder K., Schofield P., Gurland B., Andrews H., Mayeux R. (1996). Effect of oestrogen during menopause on risk and age at onset of Alzheimer’s disease. Lancet.

[66] Manly J.J., Merchant C.A., Jacobs D.M., Small S.A., Bell K., Ferin M., Mayeux R. (2000). Endogenous estrogen levels and Alzheimer’s disease among postmenopausal women. Neurology.

[67] Luine V.N. (1985). Estradiol increases choline acetyltransferase activity in specific basal forebrain nuclei and projection areas of female rats. Exp. Neurol..

[68] Shao H., Breitner J.C.S., Whitmer R.A., Wang J., Hayden K., Wengreen H., Corcoran C., Tschanz J., Norton M., Munger R. (2012). Hormone therapy and Alzheimer disease dementia: New findings from the Cache County Study. Neurology.

[69] Norbury R., Travis M.J., Erlandsson K., Waddington W., Ell P.J., Murphy D.G.M. (2007). Estrogen therapy and brain muscarinic receptor density in healthy females: A SPET study. Horm. Behav..

[70] Maki P.M. (2013). Critical window hypothesis of hormone therapy and cognition: A scientific update on clinical studies. Menopause.

[71] Shumaker S.A., Legault C., Rapp S.R., Thal L., Wallace R.B., Ockene J.K., Hendrix S.L., Jones B.N., Assaf A.R., Jackson R.D. (2003). Estrogen plus progestin and the incidence of dementia and mild cognitive impairment in postmenopausal women: The Women’s Health Initiative Memory Study: A randomized controlled trial. JAMA.

[72] Rapp S.R., Espeland M.A., Shumaker S.A., Henderson V.W., Brunner R.L., Manson J.E., Gass M.L.S., Stefanick M.L., Lane D.S., Hays J. (2003). Effect of estrogen plus progestin on global cognitive function in postmenopausal women: The Women’s Health Initiative Memory Study: A randomized controlled trial. JAMA.

[73] Silverman D.H.S., Geist C.L., Kenna H.A., Williams K., Wroolie T., Powers B., Brooks J., Rasgon N.L. (2011). Differences in regional brain metabolism associated with specific formulations of hormone therapy in postmenopausal women at risk for AD. Psychoneuroendocrinology.

[74] Lethaby A., Hogervorst E., Richards M., Yesufu A., Yaffe K. (2008). Hormone replacement therapy for cognitive function in postmenopausal women. Cochrane Database Syst. Rev..

[75] Resnick S.M., Maki P.M., Rapp S.R., Espeland M.A., Brunner R., Coker L.H., Granek I.A., Hogan P., Ockene J.K., Shumaker S.A. (2006). Effects of combination estrogen plus progestin hormone treatment on cognition and affect. J. Clin. Endocrinol. Metab..

[76] Ohayon M.M., Carskadon M.A., Guilleminault C., Vitiello M.V. (2004). Meta-analysis of quantitative sleep parameters from childhood to old age in healthy individuals: Developing normative sleep values across the human lifespan. Sleep.

[77] Tao M.F., Sun D.M., Shao H.F., Li C.B., Teng Y.C. (2016). Poor sleep in middle-aged women is not associated with menopause per se. Braz. J. Med. Biol. Res..

[78] Ameratunga D., Goldin J., Hickey M. (2012). Sleep disturbance in menopause. Intern. Med. J..

[79] Prairie B.A., Wisniewski S.R., Luther J., Hess R., Thurston R.C., Wisner K.L., Bromberger J.T. (2015). Symptoms of depressed mood, disturbed sleep, and sexual problems in midlife women: Cross-sectional data from the Study of Women’s Health Across the Nation. J. Women’s Health.

[80] Reed-Kane D. (2001). Natural hormone replacement therapy: What it is and what consumers really want. Int. J. Pharm. Compd..

[81] Hankinson S.E., Willett W.C., Manson J.E., Colditz G.A., Hunter D.J., Spiegelman D., Barbieri R.L., Speizer F.E. (1998). Plasma sex steroid hormone levels and risk of breast cancer in postmenopausal women. J. Natl. Cancer Inst..

[82] Key T., Appleby P., Barnes I., Reeves G. (2002). Endogenous sex hormones and breast cancer in postmenopausal women: Reanalysis of nine prospective studies. J. Natl. Cancer Inst..

[83] Lippert C., Seeger H., Mueck A.O. (2003). The effect of endogenous estradiol metabolites on the proliferation of human breast cancer cells. Life Sci..

[84] Gleason C.E., Dowling N.M., Wharton W., Manson J.A.E., Miller V.M., Atwood C.S., Brinton E.A., Cedars M.I., Lobo R.A., Merriam G.R. (2015). Effects of Hormone Therapy on Cognition and Mood in Recently Postmenopausal Women: Findings from the Randomized, Controlled KEEPS–Cognitive and Affective Study. PLoS Med..

[85] Cintron D., Lipford M., Larrea-Mantilla L., Spencer-Bonilla G., Lloyd R., Gionfriddo M.R., Gunjal S., Farrell A.M., Miller V.M., Murad M.H. (2017). Efficacy of menopausal hormone therapy on sleep quality: Systematic review and meta-analysis. Endocrine.

[86] Al-Safi Z.A., Santoro N. (2018). Menopausal hormone therapy and menopausal symptoms. Fertil. Steril..

[87] Høibraaten E., Abdelnoor M., Sandset P.M. (1999). Hormone Replacement Therapy with Estradiol and Risk of Venous Thromboembolism A Population-based Case-control Study. Thromb. Haemost..

[88] Canonico M., Oger E., Plu-Bureau G., Conard J., Meyer G., Levesque H., Trillot N., Barrellier M.-T., Wahl D., Emmerich J. (2007). Hormone therapy and venous thromboembolism among postmenopausal women: Impact of the route of estrogen administration and progestogens: The ESTHER study. Circulation.

[89] Canonico M., Fournier A., Carcaillon L., Olie V., Plu-Bureau G., Oger E., Mesrine S., Boutron-Ruault M.-C., Clavel-Chapelon F., Scarabin P.-Y. (2010). Postmenopausal hormone therapy and risk of idiopathic venous thromboembolism: Results from the E3N cohort study. Arterioscler. Thromb. Vasc. Biol..

[90] Simon J.A., Hsia J., Cauley J.A., Richards C., Harris F., Fong J., Barrett-Connor E., Hulley S.B. (2001). Postmenopausal hormone therapy and risk of stroke: The Heart and Estrogen-progestin Replacement Study (HERS). Circulation.

[91] Bath P.M.W., Gray L.J. (2005). Association between hormone replacement therapy and subsequent stroke: A meta-analysis. BMJ.

[92] Grodstein F., Manson J.E., Stampfer M.J., Rexrode K. (2008). Postmenopausal hormone therapy and stroke: Role of time since menopause and age at initiation of hormone therapy. Arch. Intern. Med..

[93] Renoux C., Dell’aniello S., Garbe E., Suissa S. (2010). Transdermal and oral hormone replacement therapy and the risk of stroke: A nested case-control study. BMJ.

[94] Rossouw J.E., Anderson G.L., Prentice R.L., LaCroix A.Z., Kooperberg C., Stefanick M.L., Jackson R.D., Beresford S.A.A., Howard B.V., Johnson K.C. (2002). Risks and benefits of estrogen plus progestin in healthy postmenopausal women: Principal results From the Women’s Health Initiative randomized controlled trial. JAMA.

[95] Chlebowski R.T., Rohan T.E., Manson J.E., Aragaki A.K., Kaunitz A., Stefanick M.L., Simon M.S., Johnson K.C., Wactawski-Wende J., O’sullivan M.J. (2015). Breast cancer after use of estrogen plus progestin and estrogen alone: Analyses of data from 2 women’s health initiative randomized clinical trials. JAMA Oncol..

[96] Jones M.E., Schoemaker M.J., Wright L., McFadden E., Griffin J., Thomas D., Hemming J., Wright K., Ashworth A., Swerdlow A.J. (2016). Menopausal hormone therapy and breast cancer: What is the true size of the increased risk?. Br. J. Cancer.

[97] Lowe K.A., Chia V.M., Taylor A., O’Malley C., Kelsh M., Mohamed M., Mowat F.S., Goff B. (2013). An international assessment of ovarian cancer incidence and mortality. Gynecol. Oncol..

[98] (2015). Collaborative Group on Epidemiological Studies of Ovarian Cancer Menopausal hormone use and ovarian cancer risk: Individual participant meta-analysis of 52 epidemiological studies. Lancet.

[99] Siegel R., Ward E., Brawley O., Jemal A. (2011). Cancer statistics, 2011: The impact of eliminating socioeconomic and racial disparities on premature cancer deaths. CA Cancer J. Clin..

[100] Anderson G.L., Judd H.L., Kaunitz A.M., Barad D.H., Beresford S.A.A., Pettinger M., Liu J., McNeeley S.G., Lopez A.M. (2003). Effects of estrogen plus progestin on gynecologic cancers and associated diagnostic procedures: The Women’s Health Initiative randomized trial. JAMA.

[101] Grady D., Gebretsadik T., Kerlikowske K., Ernster V., Petitti D. (1995). Hormone replacement therapy and endometrial cancer risk: A meta-analysis. Obstet. Gynecol..

[102] Sjögren L.L., Mørch L.S., Løkkegaard E. (2016). Hormone replacement therapy and the risk of endometrial cancer: A systematic review. Maturitas.

[103] MacLennan A.H. (2011). HRT in difficult circumstances: Are there any absolute contraindications?. Climacteric.

[104] Loria P., Petraglia F., Concari M., Bertolotti M., Martella P., Luisi S., Grisolia C., Foresta C., Volpe A., Genazzani A.R. (1998). Influence of age and sex on serum concentrations of total dimeric activin A. Eur. J. Endocrinol..

[105] Reame N.E., Lukacs J.L., Olton P., Ansbacher R., Padmanabhan V. (2007). Differential Effects of Aging on Activin A and its Binding Protein, Follistatin, across the Menopause Transition. Fertil. Steril..

[106] Bancroft J., Sherwin B.B., Alexander G.M., Davidson D.W., Walker A. (1991). Oral contraceptives, androgens, and the sexuality of young women: II. The role of androgens. Arch. Sex. Behav..

[107] Appelt H., Strauss B. (1984). Effects of antiandrogen treatment on the sexuality of women with hyperandrogenism. Psychother. Psychosom..

[108] Buster J.E., Kingsberg S.A., Aguirre O., Brown C., Breaux J.G., Buch A., Rodenberg C.A., Wekselman K., Casson P. (2005). Testosterone patch for low sexual desire in surgically menopausal women: A randomized trial. Obstet. Gynecol..

[109] Panay N., Al-Azzawi F., Bouchard C., Davis S.R., Eden J., Lodhi I., Rees M., Rodenberg C.A., Rymer J., Schwenkhagen A. (2010). Testosterone treatment of HSDD in naturally menopausal women: The ADORE study. Climacteric.

[110] Maclaran K., Panay N. (2011). Managing low sexual desire in women. Women’s Health.

[111] Fritz M.A., Speroff L. (2011). Clinical Gynecologic Endocrinology and Infertility.

[112] MacLaughlin D.T., Donahoe P.K. (2010). Müllerian Inhibiting Substance/anti-Müllerian hormone: A potential therapeutic agent for human ovarian and other cancers. Futur. Oncol..

[113] Kim J.H., MacLaughlin D.T., Donahoe P.K. (2014). Mullerian inhibiting substance/anti-Mullerian hormone: A novel treatment for gynecologic tumors. Obstet. Gynecol. Sci..

[114] Hwang S.J., Suh M.J., Yoon J.H., Kim M.R., Ryu K.S., Nam S.W., Donahoe P.K., Maclaughlin D.T., Kim J.H. (2011). Identification of characteristic molecular signature of Mullerian inhibiting substance in human HPV-related cervical cancer cells. Int. J. Oncol..

[115] Anttonen M., Farkkila A., Tauriala H., Kauppinen M., MacLaughlin D.T., Unkila-Kallio L., Butzow R., Heikinheimo M. (2011). Anti-Mullerian hormone inhibits growth of AMH type II receptor-positive human ovarian granulosa cell tumor cells by activating apoptosis. Lab. Investig..

[116] Pepin D., Sosulski A., Zhang L., Wang D., Vathipadiekal V., Hendren K., Coletti C.M., Yu A., Castro C.M., Birrer M.J. (2015). AAV9 delivering a modified human Mullerian inhibiting substance as a gene therapy in patient-derived xenografts of ovarian cancer. Proc. Nal. Acad. Sci. USA.

[117] Papakostas T.D., Pieretti-Vanmarcke R., Nicolaou F., Thanos A., Trichonas G., Koufomichali X., Anago K., Donahoe P.K., Teixeira J., MacLaughlin D.T. (2010). Development of an efficiently cleaved, bioactive, highly pure FLAG-tagged recombinant human Mullerian Inhibiting Substance. Protein Expr. Purif..

[118] Matzuk M.M., Finegold M.J., Su J.G., Hsueh A.J., Bradley A. (1992). Alpha-inhibin is a tumour-suppressor gene with gonadal specificity in mice. Nature.

[119] Matzuk M.M., Finegold M.J., Mather J.P., Krummen L., Lu H., Bradley A. (1994). Development of cancer cachexia-like syndrome and adrenal tumors in inhibin-deficient mice. Proc. Nal. Acad. Sci. USA.

[120] Pangas S.A., Woodruff T.K. (2002). Production and purification of recombinant human inhibin and activin. J. Endocrinol..

[121] Conti E., Musumeci M.B., Assenza G.E., Quarta G., Autore C., Volpe M. (2008). Recombinant human insulin-like growth factor-1: A new cardiovascular disease treatment option?. Cardiovasc. Hematol. Agents Med. Chem..

[122] Saukkonen T., Amin R., Williams R.M., Fox C., Yuen K.C., White M.A., Umpleby A.M., Acerini C.L., Dunger D.B. (2004). Dose-dependent effects of recombinant human insulin-like growth factor (IGF)-I/IGF binding protein-3 complex on overnight growth hormone secretion and insulin sensitivity in type 1 diabetes. J. Clin. Endocrinol. Metab..

[123] Wilson B.C., Milne P., Saleh T.M. (2005). Relaxin pretreatment decreases infarct size in male rats after middle cerebral artery occlusion. Ann. N. Y. Acad. Sci..

[124] Wilson B.C., Connell B., Saleh T.M. (2006). Relaxin-induced reduction of infarct size in male rats receiving MCAO is dependent on nitric oxide synthesis and not estrogenic mechanisms. Neurosci. Lett..

[125] Danielson L.A., Conrad K.P. (2003). Time course and dose response of relaxin-mediated renal vasodilation, hyperfiltration, and changes in plasma osmolality in conscious rats. J. Appl. Physiol..

[126] Conrad K.P., Debrah D.O., Novak J., Danielson L.A., Shroff S.G. (2004). Relaxin modifies systemic arterial resistance and compliance in conscious, nonpregnant rats. Endocrinology.

[127] Debrah D.O., Conrad K.P., Danielson L.A., Shroff S.G. (2005). Effects of relaxin on systemic arterial hemodynamics and mechanical properties in conscious rats: Sex dependency and dose response. J. Appl. Physiol..

[128] Unemori E.N., Lewis M., Constant J., Arnold G., Grove B.H., Normand J., Deshpande U., Salles A., Pickford L.B., Erikson M.E. (2000). Relaxin induces vascular endothelial growth factor expression and angiogenesis selectively at wound sites. Wound Repair Regen..

[129] Masini E., Salvemini D., Mugnai L., Bello M.G., Bani D., Mannaioni P.F. (1996). The effect of relaxin on myocardial ischaemia-reperfusion injury and histamine release in vitro and in vivo. Inflamm. Res..

[130] Masini E., Bani D., Bello M.G., Bigazzi M., Mannaioni P.F., Sacchi T.B. (1997). Relaxin counteracts myocardial damage induced by ischemia-reperfusion in isolated guinea pig hearts: Evidence for an involvement of nitric oxide. Endocrinology.

[131] Bani D., Masini E., Bello M.G., Bigazzi M., Sacchi T.B. (1998). Relaxin protects against myocardial injury caused by ischemia and reperfusion in rat heart. Am. J. Pathol..

[132] Bani D., Bigazzi M., Masini E., Bani G., Sacchi T.B. (1995). Relaxin depresses platelet aggregation: In vitro studies on isolated human and rabbit platelets. Lab. Investig..

[133] Nistri S., Chiappini L., Sassoli C., Bani D. (2003). Relaxin inhibits lipopolysaccharide-induced adhesion of neutrophils to coronary endothelial cells by a nitric oxide-mediated mechanism. FASEB J..

[134] Masini E., Nistri S., Vannacci A., Bani Sacchi T., Novelli A., Bani D. (2004). Relaxin inhibits the activation of human neutrophils: Involvement of the nitric oxide pathway. Endocrinology.

[135] Bani D., Ballati L., Masini E., Bigazzi M., Sacchi T.B. (1997). Relaxin counteracts asthma-like reaction induced by inhaled antigen in sensitized guinea pigs. Endocrinology.

[136] Mookerjee I., Tang M.L.K., Solly N., Tregear G.W., Samuel C.S. (2005). Investigating the role of relaxin in the regulation of airway fibrosis in animal models of acute and chronic allergic airway disease. Ann. N. Y. Acad. Sci..

[137] Wyatt T.A., Sisson J.H., Forget M.A., Bennett R.G., Hamel F.G., Spurzem J.R. (2002). Relaxin stimulates bronchial epithelial cell PKA activation, migration, and ciliary beating. Exp. Biol. Med..

[138] Teerlink J.R., Cotter G., Davison B.A., Felker G.M., Filippatos G., Greenberg B.H., Ponikowski P., Unemori E., Voors A.A., Adams K.F. (2013). Serelaxin, recombinant human relaxin-2, for treatment of acute heart failure (RELAX-AHF): A randomised, placebo-controlled trial. Lancet.

[139] Dschietzig T.B. (2014). Recombinant human relaxin-2: (How) can a pregnancy hormone save lives in acute heart failure?. Am. J. Cardiovasc. Drugs.

[140] Longcope C., Gorbach S., Goldin B., Woods M., Dwyer J., Warram J. (1985). The metabolism of estradiol; oral compared to intravenous administration. J. Steroid Biochem..

[141] Serin I.S., Ozcelik B., Basbug M., Aygen E., Kula M., Erez R. (2001). Long-term effects of continuous oral and transdermal estrogen replacement therapy on sex hormone binding globulin and free testosterone levels. Eur. J. Obstet. Gynecol. Reprod. Biol..

[142] Stomati M., Hartmann B., Spinetti A., Mailand D., Rubino S., Albrecht A., Huber J., Petraglia F., Genazzani A.R. (1996). Effects of hormonal replacement therapy on plasma sex hormone-binding globulin, androgen and insulin-like growth factor-1 levels in postmenopausal women. J. Endocrinol. Investig..

[143] Krotz S.P., Robins J.C., Ferruccio T.-M., Moore R., Steinhoff M.M., Morgan J.R., Carson S. (2010). In vitro maturation of oocytes via the pre-fabricated self-assembled artificial human ovary. J. Assist. Reprod. Genet..

[144] Sittadjody S., Saul J.M., Joo S., Yoo J.J., Atala A., Opara E.C. (2013). Engineered multilayer ovarian tissue that secretes sex steroids and peptide hormones in response to gonadotropins. Biomaterials.

[145] Liu C., Xia X., Miao W., Luan X., Sun L., Jin Y., Liu L. (2013). An ovarian cell microcapsule system simulating follicle structure for providing endogenous female hormones. Int. J. Pharm..

[146] Guo X.-X., Zhou J.-L., Xu Q., Lu X., Liang Y.-J., Weng J., Shi X.-L. (2010). Prevention of osteoporosis in mice after ovariectomy via allograft of microencapsulated ovarian cells. Anat. Rec..

[147] Merchenthaler I., Lane M., Sabnis G., Brodie A., Nguyen V., Prokai L., Prokai-Tatrai K. (2016). Treatment with an orally bioavailable prodrug of 17β-estradiol alleviates hot flushes without hormonal effects in the periphery. Sci. Rep..

[148] Prokai L., Nguyen V., Szarka S., Garg P., Sabnis G., Bimonte-Nelson H.A., McLaughlin K.J., Talboom J.S., Conrad C.D. (2015). The prodrug DHED selectively delivers 17β-estradiol to the brain for treating estrogen-responsive disorders. Sci. Transl. Med..

[149] Cargill S.L., Carey J.R., Muller H.-G., Anderson G. (2003). Age of ovary determines remaining life expectancy in old ovariectomized mice. Aging Cell.

[150] Mason J.B., Cargill S.L., Anderson G.B., Carey J.R. (2009). Transplantation of young ovaries to old mice increased life span in transplant recipients. J. Gerontol. Ser. A.

[151] Shikanov A., Zhang Z., Xu M., Smith R.M., Rajan A., Woodruff T.K., Shea L.D. (2011). Fibrin encapsulation and vascular endothelial growth factor delivery promotes ovarian graft survival in mice. Tissue Eng. Part A.

[152] Shea L.D., Woodruff T.K., Shikanov A. (2014). Bioengineering the ovarian follicle microenvironment. Annu. Rev. Biomed. Eng..

[153] Silber S.J. (2012). Ovary cryopreservation and transplantation for fertility preservation. Mol. Hum. Reprod..

[154] Oktay K., Turkcuoglu I., Rodriguez-Wallberg K.A. (2011). Four spontaneous pregnancies and three live births following subcutaneous transplantation of frozen banked ovarian tissue: What is the explanation?. Fertil. Steril..

